# An Electrophysiological Abstractness Effect for Metaphorical Meaning Making

**DOI:** 10.1523/ENEURO.0052-20.2020

**Published:** 2020-09-10

**Authors:** Bálint Forgács

**Affiliations:** 1 Laboratoire Psychologie de la Perception (LPP), Université de Paris, Paris 75006, France; 2 MTA-ELTE Social Minds Research Group, Eötvös Loránd University (ELTE), Budapest 1064, Hungary

**Keywords:** concreteness effect, EEG, embodiment, figurative language, metaphor, novel language

## Abstract

Neuroimaging studies show that metaphors activate sensorimotor areas. These findings were interpreted as metaphors contributing to conceptual thought by mapping concrete, somatosensory information onto abstract ideas. But is sensorimotor information a necessary constituent of figurative meaning? The present study employed event-related potentials (ERPs) in a divided visual field paradigm with healthy adults to explore the role of sensorimotor feature processing in the comprehension of novel metaphors via the electrophysiological concreteness effect. Participants read French, novel adjective-noun expressions that were either metaphorical (“fat sentence”) or literal (“fat hip”). While literal expressions evoked a typical concreteness effect, an enhanced frontal negativity during right hemisphere (RH) as opposed to left hemisphere (LH) presentation, metaphors showed no such sign of sensorimotor feature processing. Relative to literals, they evoked a sustained frontal negativity during LH presentation and similar amplitudes during RH presentation, but both of these effects were the greater the more abstract the metaphors were. It is the first time such an electrophysiological abstractness effect is reported, just the opposite of a concreteness effect. It is particularly noteworthy that ERPs evoked by metaphors were not contingent on figurativeness, novelty, meaningfulness, imageability, emotional valence, or arousal, only on abstractness. When compared with similarly novel literal expressions, metaphors did not evoke a typical N400 and did not activate the RH either. The findings shed new light on the neurocognitive machinery of figurative meaning construction, pervasive in everyday communication. Contrary to embodied cognition, the conceptual system might be organized around abstract representations and not sensorimotor information, even for lush, metaphorical language.

## Significance Statement

In the past decades, several popular theories have been promoting the idea that the format of semantic representation is sensomotoric and/or based on experience. The abstractness effect reported here challenges such Empiricist accounts of the conceptual system, like embodiment or connectionism. It argues against metaphors being the vehicles of transmitting sensorimotor information toward higher domains of cognition. Instead, even perceptual metaphors appear to evoke neural responses contingent on their abstractness. The current findings also challenge long held notions about the central role of the right cerebral hemisphere or literal meaning in comprehending figures of speech and suggest instead that the brain is not sensitive to figurativeness per se but to the abstractness it brings along.

## Introduction

Metaphor has been a small but crucial area of study in the cognitive neuroscience of language, and some scholars consider it to be an essential feat of human cognition (Lakoff and Johnson, 1980; Mithen, 1996; Pinker, 2010). The aim of the current study was to test the role of literal meaning, or sensorimotor processes, as embodiment frames it (Lakoff and Johnson, 1999), in their comprehension.

Metaphors are often described as vivid and imagistic conceptual tools and have been proposed to rely on sensorimotor computations (Gallese and Lakoff, 2005; Lakoff, 2014). Several functional magnetic resonance imaging (fMRI) studies found that metaphors activate sensorimotor brain regions for motion (Boulenger et al., 2009, 2012), texture (Lacey et al., 2012), taste (Citron and Goldberg, 2014), smell (Pomp et al., 2018), and motion sensitive visual areas or their vicinity for action (Chen et al., 2008; Saygin et al., 2010; Desai et al., 2011; Cardillo et al., 2012; Lacey et al., 2017). However, as Mahon and Caramazza (2008) pointed out, the activation of sensorimotor areas could be because of automatic spreading activation, which could have driven fMRI results without any causal role.

It has been argued that sensorimotor processes play a critical role in word comprehension because of their early involvement (∼200 ms; Pulvermüller, 2005; Pulvermüller et al., 2005). Early automatic activations of alternative meanings of ambiguous words, however, are suppressed by 200–300 ms (Swinney, 1979; Seidenberg et al., 1982; Gergely and Pléh, 1994), just as motor activations for action words (Shtyrov et al., 2014). Therefore, literal senses of metaphors and corresponding sensorimotor activations could be suppressed during lexical access. Schneider et al. (2014)’s metaphor study found a single connection between event-related potentials (ERPs) and BOLD signals in an early, 200- to 270-ms time window, which raise the possibility that fMRI studies may not have reported processes beyond early (automatic, spreading) sensorimotor activations for figurative meaning.

Another indicator of sensorimotor processes is the electrophysiological concreteness effect. It can be evoked by concrete (e.g., “chair”) as opposed to abstract words (e.g., “truth”) and has two negative frontal components: the concreteness N400, reflecting the activation of a larger set of perceptual semantic features, and the N700, thought to indicate mental imagery (Kounios and Holcomb, 1994; Holcomb et al., 1999; West and Holcomb, 2000), specific to the right hemisphere (RH) (Huang et al., 2010). Barber et al. (2013) showed that concreteness effects can be evoked even when concrete and abstract words are matched in terms of imageability and semantic richness: they propose that the frontal N400 reflects the activation of multimodal features, while the N700 is related to the integration of such sensorimotor features into a representation.

Only a couple of studies looked into the sensorimotor processing of metaphors. Two of them reported an enhanced N400 response, an indicator of demanding semantic processing (Kutas and Federmeier, 2011; Brouwer et al., 2012). However, one did not balance stimuli for familiarity and imageability (Schmidt-Snoek et al., 2015), while the other did not match metaphorical and literal conditions in terms of novelty (Shen et al., 2015), thus the reported effects might be driven by novelty instead of figurativeness. One study attempted to control for novelty employing target nouns that combined into unfamiliar metaphorical, and concrete and abstract literal expressions (Forgács et al., 2015). The authors found that only less concrete (i.e. abstract) metaphors evoked a greater negativity in the N400 time window than abstract expressions, more concrete metaphors, paradoxically, did not.

Several groups reported typical centro-parietal N400 effects for novel metaphors, and interpreted them as reflecting mappings (Coulson and Van Petten, 2002; Rutter et al., 2012; Lai and Curran, 2013; Schneider et al., 2014; Rataj et al., 2018) or as the activation of literal senses (Pynte et al., 1996; Tartter et al., 2002; Weiland et al., 2014). In lack of matched novel literal conditions, however, these studies could have reported mere novelty N400 effects (cf. Davenport and Coulson, 2013).

The goal of the present experiment is to investigate the role of sensorimotor feature processing in the comprehension of novel perceptual metaphors via the electrophysiological concreteness effect. The following predictions were made. According to strong embodiment, metaphors should not differ from literal expressions in terms of the frontal N400 and N700 concreteness effects during RH presentation. If, however, metaphors are comprehended without sensorimotor computations (besides early automatic activations), they should evoke a reduced N700 in the RH. Once controlled for novelty, metaphors should neither evoke a typical N400 effect relative to literal expressions nor activate the RH relative to the LH.

## Materials and Methods

### Participants

Thirty-six healthy adults (24 female) between ages 18–35 (M =* *25, SD =* *4) took part in the experiment. Participants were recruited through the RISC website (http://www.risc.cnrs.fr) and received €15 compensation for their work. They were all native speakers of French, right handed as measured by the Edinburgh Handedness Inventory (Oldfield, 1971) scoring above 75 points (M =* *92.1, SD =* *9.21), had normal or corrected to normal vision, and reported no neurologic or psychiatric problems. Sample size was determined based on prior research (Lee and Federmeier, 2008; Huang et al., 2010; Forgács et al., 2015). An additional eight participants were excluded from statistical analyses because of excessive blink, eye-movement, and other ERP artifacts.

### Stimuli

The study was conducted in French, where, as in Latin languages, adjectives typically follow nouns, which allows for measuring neural responses right at the adjective: the concrete, physical vehicle of the metaphor. An initial set of 320 word triplets were generated where a physical adjective, which refers to a perceptual experience (e.g., “tasty”) modified two nouns to form a metaphorical (e.g., “tasty dependence”) and a literal expression (“tasty plum”). Care was taken to make sure that none of the metaphors had a possible literal interpretation. The bigram frequency of metaphorical and literal word pairs was kept below 20 hits in a Google search of the French web to ensure that the expressions were novel, as novel metaphors are more likely to evoke sensorimotor (Binder and Desai, 2011) and RH processes (Bohrn et al., 2012). Adjectives were maximum 11 characters long (M =* *7.42, SD =* *1.67) to aid readability during lateralized presentation. The word pairs were rated in a norming study by 103 volunteers recruited via the RISC website, who received a €10/h compensation, and did not take part in the EEG experiment. Participants rated the expressions on seven-point Likert scales along three randomly assigned tasks for meaningfulness (“How meaningful is it?”), concreteness (“How easy is it to experience with the senses?”), and to avoid the definition or explanation of the notion of *metaphorical*, literalness (“How literal is it?”). The best 200 word triplets (forming two expressions) were selected with the highest meaningfulness values (at least 2.5), with the concreteness of the metaphor being lower than that of its literal counterpart (41 items of the original 320 showed an inverse pattern and were excluded), and the literalness of the metaphor being lower than that of its literal counterpart (an additional 62 items showed the inverse and were excluded). The final 200 items were rated in a second norming study by 63 volunteers (with the same conditions as above) on a seven-point Likert scale according to imageability (“How imaginable is it?”), valence (“What is its emotional valence?”), arousal (“How arousing is it?”), and the concreteness of the noun (“How easy is it to experience with the senses?”). Pairwise comparisons using paired *t* tests, and an independent sample *t* test for noun concreteness, revealed significant differences for all variables but bigram frequency and arousal. Metaphors were slightly less meaningful (still around the median of the scale), less literal, concrete, imageable, and slightly more emotionally negative than literal counterparts. Nouns in literal expressions were more concrete than in metaphorical expressions. Norming results are presented in Table 1. The full stimulus list can be viewed in Extended Data Table 1-1.

**Table 1:** Psycholinguistic properties and differences of the stimuli (*N* = 200)

	Metaphors mean (SD)	Literals mean (SD)	Levene’s *F* value	*t* value/ *S* value	95% confidence interval
Bigram frequency	3.81 (4.36)	4.06 (4.29)	0.293	0.652	[–0.496, 0.986]
Meaningfulness	4.04 (0.92)	5.39 (0.95)	0.009	15.8^***^	[1.18, 1.51]
Literalness	3.37 (0.75)	5.11 (0.86)	3.92^*^	200^***^	[1.40, 1.85]
Concreteness	2.80 (0.68)	4.49 (0.92)	21.7^***^	200^***^	[1.44, 1.81]
Imageability	4.06 (0.66)	5.28 (0.67)	0.018	19.5^***^	[1.09, 1.34]
Valence (±3)	–0.84 (1.15)	–0.66 (1.27)	3.63	2.53^*^	[0.038, 0.309]
Arousal	3.81 (0.83)	3.71 (0.88)	0.550	–1.87	[–0.202, 0.005]
Noun concreteness	2.49 (0.72)	4.29 (0.73)	3.89^*^	191^***^	[1.57, 1.92]

When the variance of a measure was not equal between metaphors and literals, as reported by a median centered Levene’s test (*F* value), a sign test (*S* value) was used, otherwise a *t* test (*t* value). Metaphorical and literal expressions differed on all properties except for their bigram frequency and arousal value. The full stimulus list is shown in Extended Data Table 1-1.

* *p *<* *0.05.

*** *p *<* *0.001.

10.1523/ENEURO.0052-20.2020.t1-1 Extended Data Table 1-1 Complete stimulus list. Every adjective was paired with two nouns to form both a metaphorical and a literal expression. Each participant saw either the literal or the figurative pairing for one particular adjective. Download Table 1-1, DOC file.

### Experimental procedures

Huang et al. (2010)’s word pair paradigm has been adopted, which measured ERPs using the divided visual field technique, to compare identical target words both in metaphorical and literal constructs, and to avoid confounding sentence processing effects. The experiment was approved by the Ethics Committee of Université de Paris, and participants gave their written informed consent on arrival to the lab. They were seated in a dimly lit room 60 cm from a screen with their head placed on a chinrest. Their task was to read word pairs (“scarified history”) followed by a probe word (“amnesia”) and to decide, using a button box, whether the probe was semantically related to the combined meaning of the preceding two-word expression or not. Such a procedure was adapted to make sure participants read for comprehension. They read the instructions and completed 16 practice trials before the experiment started. Stimuli was presented on a black background in 28-point Arial capital letters. Each trial started with four plus signs “++++” (1000 ms), after which a blank screen appeared with a jitter (800–1200 ms). Next, the prime word (noun) appeared centrally for 200 ms, and after a jittered blank screen (300–400 ms), the target word (adjective) was presented for 200 ms either to the left or right visual field. The inner most edge of target words were 1.5° visual angle away from the center of the screen. After a blank screen (1300 ms), the probe word appeared (200 ms), which was followed by another blank screen (800 ms) and a question mark “?”, which remained on the screen until participants responded. Except during the question mark a small red dot was presented centrally and slightly below the words; participants were requested to fixate it during lateralized presentation and to try not to blink when it was visible. The experiment took ∼35–40 min with short breaks between the five blocks of stimuli, each consisting of 40 trials. Participants were allowed to take short blink breaks if necessary also when the question mark was present. Each individual was assigned an individual pseudo-randomized stimulus file, with no more than three consecutive trials in either visual fields, and no more than two consecutive word pairs from the same condition. Stimulus presentation and EEG triggering was controlled via E-prime 2.08 software (Psychology Software Tools). All raw EEG data, stimulus code, analysis scripts, and full statistical results are available at http://osf.io/j45gt/.

### EEG recording and analysis

**Figure 1.**
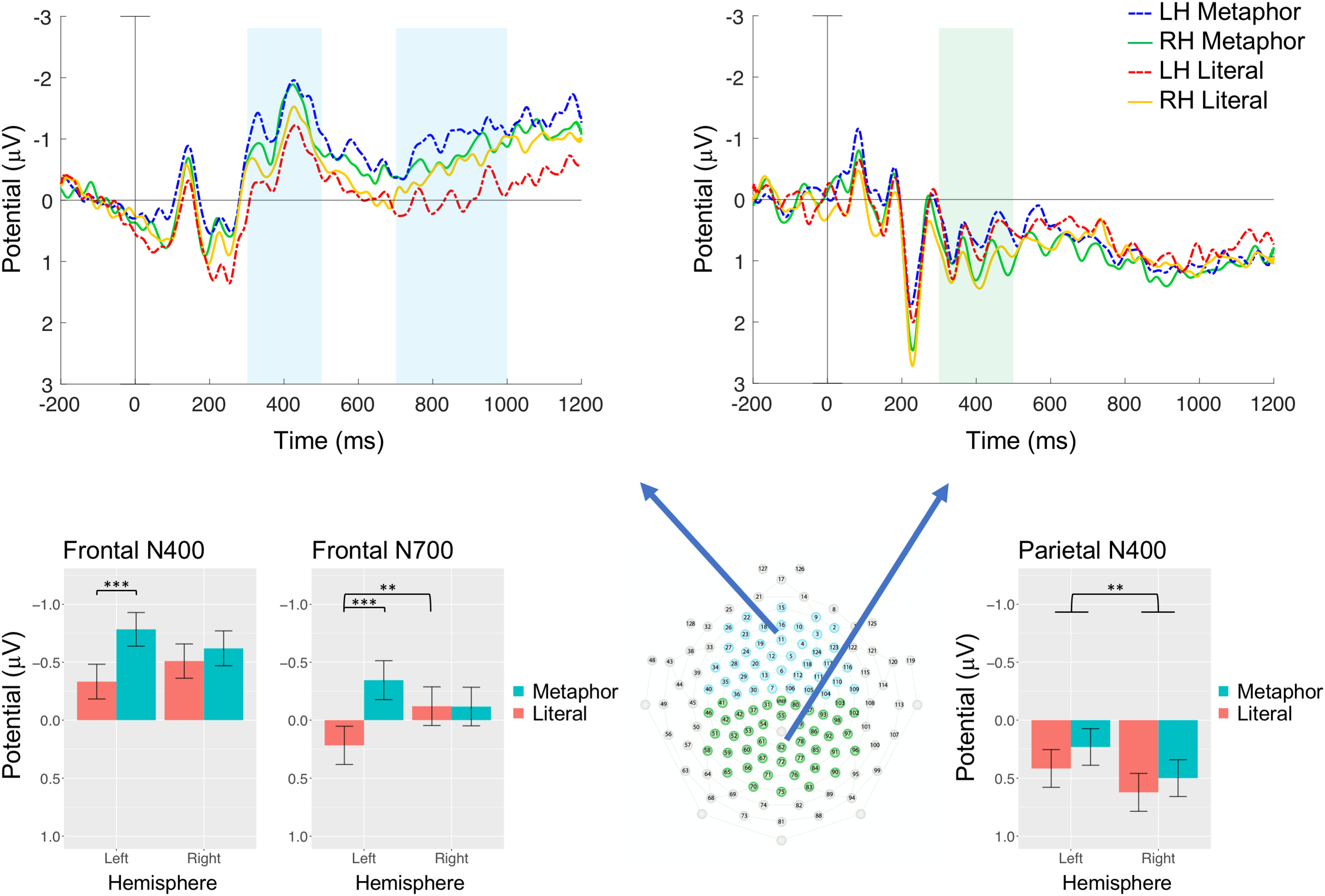
ERP responses on a frontal (Fz) and a parietal (Pz) exemplar electrode (upper left and right panels, respectively). Blue shades indicate the N400 (300–500 ms) and the N700 (700–1000 ms), the green shade the N400 time window. Negative is plotted upwards. Lower panels show bar charts of amplitudes averaged within the predifined time windows and over electrodes in the frontal and parietal Regions-of-Interest, which are shown in the middle. Error bars indicate 95% confidence intervals (**p < 0.01, ***p < 0.001). Metaphors evoked an enhanced frontal negativity relative to Literals both in the N400 (300–500 ms) and the N700 (700–1000 ms) time windows during right visual field / left hemisphere (LH) presentation. This effect was the more enhanced the more abstract the metaphors were, and it is unique and specific to the processing of metaphorical senses of concrete words (“sweet”); no such effect has been reported before for novel metaphors or non-sentential stimuli. Literals evoked a greater frontal negativity in the N700 time window during right hemisphere (RH) relative to LH presentation, which corresponds to a typical concreteness effect. Both metaphorical and literal novel word pairs evoked a greater parietal negativity in the N400 time window during LH compared with RH presentation: contrary to some prominent language lateralization models, novel expressions engaged the LH more than the RH. Grand-average ERP plots over all electrode sites for LH and RH presentation are provided in Extended Data Figures 1-1, 1-2, respectively. Electrophysiological evidence of successful lateralized presentation of target words is presented in Extended Data Figure 1-3.

The EEG signal was recorded continuously with EGI’s 128-channel HydroCel Geodesic Sensor Net at 500-Hz sampling rate. Electrode impedance was kept below 50 kΩ and readjusted during breaks when necessary. The EEG recording was analyzed with Net Station 4.5.6 (Electrical Geodesics Inc.). Raw EEG data were filtered using a 0.3-Hz high-pass and a 30-Hz low-pass filter and segmented into epochs 200 ms before and 1200 ms after the onset of target words. Automatic artifact detection algorithms for blinks, eye-movements, and bad channels were used to reject bad segments, which was confirmed via visual inspection. Bad channels were replaced by spherical spline interpolation, and the data was baseline-corrected to the 200-ms preceding word onset and re-referenced to the average reference. Participants needed to produce at least 30 clean trials per condition per visual field to be included in the final statistical tests. On average, participants contributed 43 trials to each of the four conditions (86%). For the typical N400 effect, mean amplitudes were extracted between 300–500 ms (Kutas and Federmeier, 2011) and averaged over a parietal region of interest (ROI) that included the following electrodes: 31, 37, 41, 42, 46, 47, 51, 52, 53, 54, 55, 58, 59, 60, 61, 62, 65, 66, 67, 70, 71, 72, 75, 76, 77, 78, 79, 80, 83, 84, 85, 86, 87, 90, 91, 92, 93, 96, 97, 98, 102, 103, VREF (Fig. 1). Concreteness effect ERP responses were analyzed over a frontal ROI (that included the following electrodes: 2, 3, 4, 5, 6, 7, 9, 10, 11, 12, 13, 15, 16, 18, 19, 20, 22, 23, 24, 26, 27, 28, 29, 30, 34, 35, 36, 40, 104, 105, 106, 109, 110, 111, 112, 116, 117, 118, 123, 124) in the 300- to 500-ms and 700- to 1000-ms time windows, following Lucas et al. (2017), who used novel conceptual combinations to study concreteness.

10.1523/ENEURO.0052-20.2020.f1-1 Extended Data Figure 1-1 Grand-average ERP plots for LH presentation over each electrode site. Negative is plotted upwards, frontal sites are above, parietal sites are below. Black line is the literal, red line is the metaphorical condition. A frontal negativity is apparent between 300–500 ms and 700–1000 ms as well. Download Figure 1-1, TIF file.

**Figure 2.**
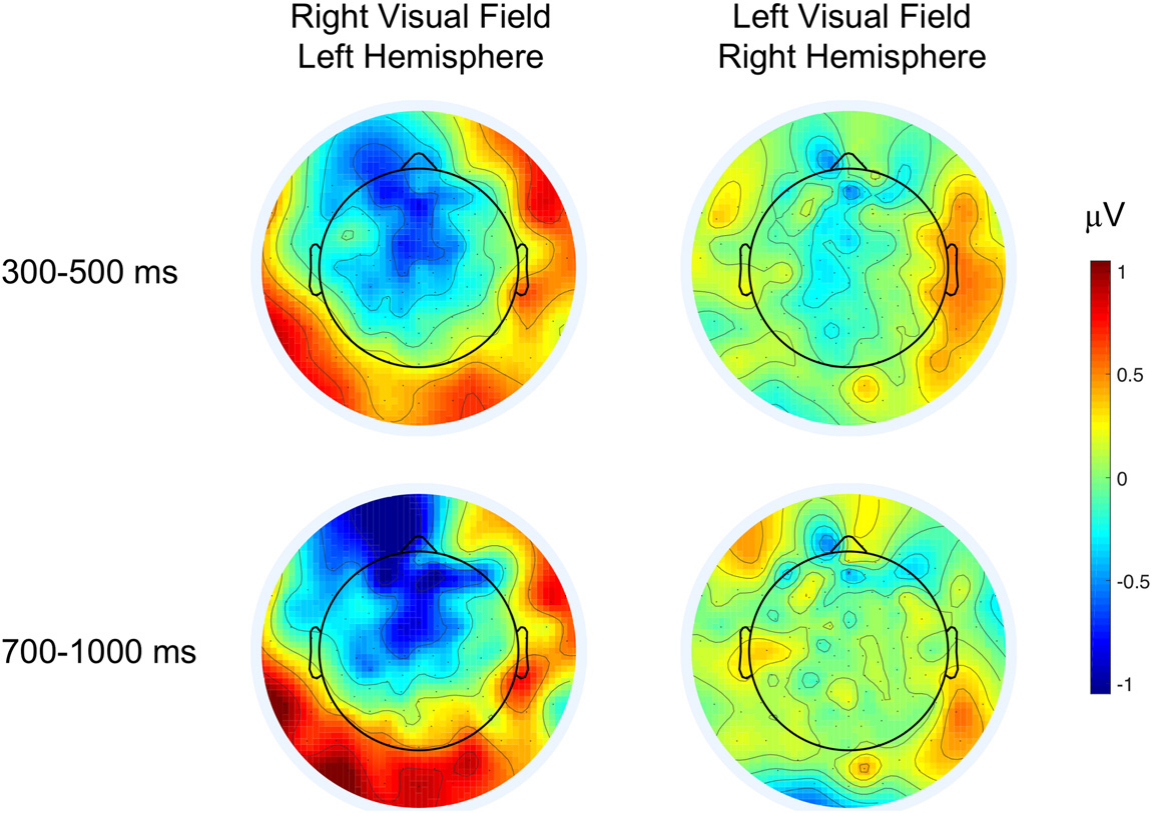
Topographical difference maps (metaphor–literal) of ERP responses in the N400 and N700 time windows for each hemifield presentation. Blue colors indicate greater negativities for metaphors, relative to literals, which is apparent in both time windows over frontal areas during LH presentation. Extended Data Figure 2-1 shows topographical maps of the frontal effects in the side contrast (RH–LH) and demonstrates a typical concreteness for literal expressions in the N700 time window. Extended Data Figures 2-2, 2-3 show effects evoked at the prime word (noun).

10.1523/ENEURO.0052-20.2020.f1-2 Extended Data Figure 1-2 Grand-average ERP plots for RH presentation over each electrode site. Negative is plotted upwards, frontal sites are above, parietal sites are below. Black line is the literal, red line is the metaphorical condition. No difference is apparent frontally between 300–500 or 700–1000 ms either. Download Figure 1-2, TIF file.

**Figure 3.**
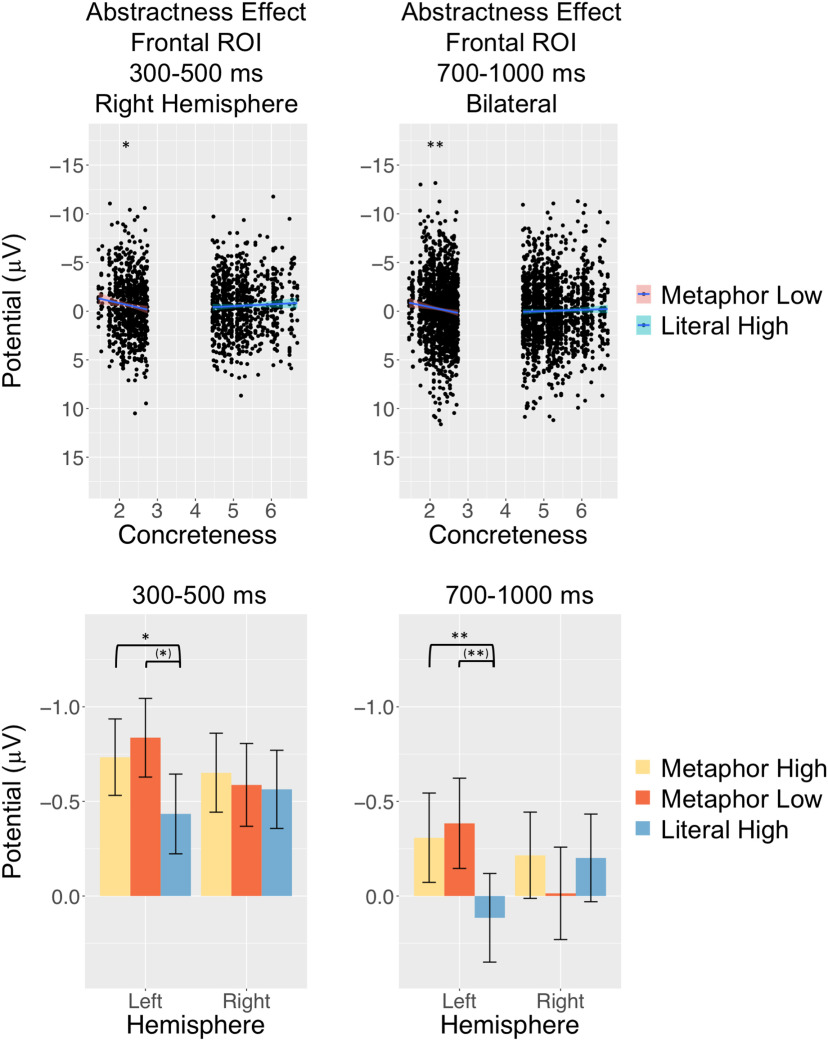
Abstractness effect evoked by metaphors over the frontal ROI. Upper panel shows ERP responses for individual trials to low-concreteness metaphors and high-concreteness literals. Electrophysiological responses over frontal electrode sites were the more negative, the less concrete (i.e., more abstract) the metaphors were both in in the 300- to 500-ms time window during RH presentation and also in the 700- to 1000-ms time window bilaterally. Lower panel shows bar charts of the median split high-concreteness and low-concreteness metaphors relative to median split high-concreteness literals. Stars in brackets (*) indicate effects that were significant if concreteness was not included in the models (i.e., concreteness accounted for these effects). Error bars indicate 95% confidence intervals (*p < 0.05, **p < 0.01).

10.1523/ENEURO.0052-20.2020.f1-3 Extended Data Figure 1-3 Markers of lateralized processing. Topographical maps in the upper row show the difference of RH–LH presentation: cold colors indicate greater negativities for RH (left visual field), warm colors for LH (right visual field) presentation. Upper left represents the N1 for literal word pairs, and upper right the selection negativity (SN) for metaphors. Lower row ERP waveforms show responses on a left and a right exemplar electrode. Red shades indicate the 100- to 200 ms (N1) time window, blue shades the 300- to 1000-ms (SN) time window. Negative is plotted upwards. Download Figure 1-3, TIF file.

### Statistical analyses

All statistical tests reported here were conducted over single trials using linear mixed-effects modeling (LMEM: Baayen, 2008; Baayen et al., 2008), with the statistical language R (Core Team R, 2017) and the lme4 package (Bates et al., 2015). Data points >2.5 SD away from each individual’s mean ERPs were removed. The following steps were taken during model building. First, the order of trials was introduced as a fixed effect against a model of random effects only, to check whether responses were modulated by fatigue; it was included only if it significantly improved the model. Presentation side (RH and LH) and word pair category (metaphor and literal) were entered in the models as fixed effects in interaction; participants and items were entered as random effects, with random slopes and intercepts for side, category, and their interaction, to keep it maximal (Barr et al., 2013). If the random effect structure was too complex for the model to converge, it was simplified stepwise. To control for possible confounds and specify the role of psycholinguistic variables, they were included in the statistical models as covariates. They were entered separately to avoid collinearity, as some were strongly correlated. The effect of emotional factors was checked by adding side × valence and side × arousal interaction terms only, since they were not expected to affect the two conditions differentially. Next, the logarithm of bigram frequency and semantic covariates (meaningfulness, concreteness, imageability and literalness) were introduced one-by-one to the model extending the side × category interaction into a three-way interaction. Covariates were included in the final model only if they improved it significantly (and otherwise are not reported). Models were compared using likelihood ratio tests, and *p* values of the final models were calculated based on the Kenward–Roger approximation with the mixed() function (Singmann and Kellen, 2017). Likelihood tests for model building are not reported in the main text but are fully available at http://osf.io/j45gt/. Model residual plots did not exhibit visible deviations from normality and homoscedasticity.

## Results

### Behavioral results

Response accuracy was highly variable across individuals but similar for related (M =* *68%, SD =* *47%) and unrelated probe words (M =* *65%, SD =* *48%), which suggests that participants payed attention and made an effort at interpreting the two-word expressions by linking their combined meaning to the probe words.

### Confirmatory ERP analyses

Frontal and parietal electrophysiological responses are shown over two exemplar electrodes together with mean amplitudes in selected ROIs and time windows in Figure 1 (grand average ERPs at each electrode site can be viewed in Extended Data Figs. 1-1, 1-2 for LH and RH presentation, respectively). Statistical analyses of two indicators of lateralized processing of visual stimuli, the N1 (100–200 ms) and the selection negativity (300–1000 ms), confirmed that lateralized presentation was successful in this study (Extended Data Fig. 1-3 and in full detail at http://osf.io/j45gt/).

#### Typical N400

Mean amplitudes were calculated in the 300- to 500-ms time window over electrodes in the parietal ROI. The final model [n400.post.lmem = mixed(n400 ∼ trial + category*side + (1 + category|participant) + (1 + side|item_nr), n400.post.data)] revealed no significant difference between the two categories (β = –0.07, SE =* *0.04, *F*_(1,34.4)_ = 3.37, *p *= 0.075) only between the two presentation sides (β = 0.12, SE =* *0.04, *F*_(1,197)_ = 8.44, *p *=* *0.004), with no interaction (β = 0.01, SE =* *0.04, *F*_(1,5892)_ = 0.11, *p *=* *0.74). Metaphors did not evoke a typical N400 relative to literals, and both conditions evoked a greater negativity during LH presentation than RH presentation (Figs. 1, 2). One participant only had 29 trials in one of the bins for the typical N400 analysis, but the exclusion of this individual did not change the pattern of results: only side had a significant main effect (*p *=* *0.002), category did not (*p *=* *0.11), and there was no interaction (*p *=* *0.84; Bonferroni corrected α = 0.025).

10.1523/ENEURO.0052-20.2020.f2-1 Extended Data Figure 2-1 Topographical maps of the frontal effects in the side contrast (RH–LH). Cold colors indicate greater negativities for RH (left visual field) presentation, warm colors for LH (right visual field) presentation. In the 300- to 500-ms time window neither metaphors (*p *= 0.11) nor literals (*p *= 0.13) were processed differently during lateralized presentation. In the N700 time window, literals evoked a greater negativity in the right than in the LH (β = –0.17, SE = 0.06, *F*_(1,2913)_ = 8.63, *p *=* *0.003), while metaphors evoked the same amount of brain electricity in both hemispheres (*p *=* *0.059; Bonferroni corrected α-level = 0.0125). Download Figure 2-1, TIF file.

10.1523/ENEURO.0052-20.2020.f2-2 Extended Data Figure 2-2 Grand-average topo plots of ERPs at the prime word (noun) at each electrode site. Nouns were presented centrally for 200 ms followed by a blank screen jittered between 300 and 400 ms. Onset of visual word presentation is at 0, it is preceded by a 200-ms baseline and followed by 500-ms analysis window. Download Figure 2-2, TIF file.

10.1523/ENEURO.0052-20.2020.f2-3 Extended Data Figure 2-3 Upper row shows ERP responses at two exemplar electrodes (Fz and Pz), lower row shows a topo map of ERPs in the N400 time window (300–500 ms) to the prime word (noun). Nouns evoked a typical N400 response, confirmed by a paired *t* test on participant averaged data over the parietal ROI (*M_diff_* = 0.39 μV, *SD_diff_* = 0.41 μV), *t*_(37)_ = 5.82, *p *< 0.001, 95% CI [0.25, 0.53 μV], Hedges’ *g*_av_ = 0.43. Such an effect could be due to a variety of psycholinguistic variables (e.g., frequency, length, neighborhood size, etc.), since prime nouns were allowed to vary on properties, but target adjectives were fully controlled. Expressions in the literal and metaphorical conditions ended on the same adjectives, thus each participant saw the same set of target words. The response at the noun is not likely to have influenced outcomes at the adjective. If it influenced the baseline for adjectives, then responses to literals should have been artefactually reduced, which should have yielded an enhanced N400 response to metaphors (which was not the case). Download Figure 2-3, TIF file.

#### Frontal N400

For the frontal N400 mean amplitudes were calculated in the 300- to 500-ms time window over the frontal ROI (Figs. 1, 2). The final model [n400.ant.lmem = mixed(n400 ∼ trial + category*side + (1|participant) + (1|item_nr), n400.ant.data)] revealed a category main effect (β = –0.15, SE =* *0.04, *F*_(1,5986)_ = 15.4, *p *<* *0.001) but also an interaction between side and category (β = 0.08, SE =* *0.04, *F*_(1,6003)_ = 4.91, *p *=* *0.027). When the interaction was broken down by presentation side, metaphors evoked a greater negativity than literals during LH presentation (β = –0.23, SE =* *0.05, *F*_(1,2917)_ = 19.4, *p *<* *0.001) but not during RH presentation (β = –0.06, SE =* *0.05, *F*_(1,2958)_ = 1.43, *p *=* *0.23).

#### Frontal N700

Over the frontal ROI mean amplitudes were calculated between 700 and 1000 ms for the N700 (Figs. 1, 2). Trial and emotional valence significantly improved the final model [n700.ant.lmem = mixed(n700 ∼ trial + category*side + side*valence + (1 + category|participant) + (1|item_nr), n700.ant.data)]. There was a significant main effect of category (β = –0.16, SE =* *0.04, *F*_(1,35.0)_ = 13.5, *p *<* *0.001), and of valence (β = –0.12, SE =* *0.04, *F*_(1,290)_ = 11.3, *p *<* *0.001), and a category × side interaction (β = 0.14, SE =* *0.04, *F*_(1,5990)_ = 11.2, *p *<* *0.001). Breaking the interaction down by side showed that metaphors induced a greater negativity than Literals during LH presentation (β = –0.29, SE =* *0.06, *F*_(1,34.9)_ = 22.2, *p *<* *0.001) but not during RH presentation (β = –0.02, SE =* *0.06, *F*_(1,2978)_ = 0.10, *p *=* *0.75).

### Exploratory analyses of frontal ERPs

It is a question whether literal expressions evoked a concreteness effect. Huang et al. (2010) reported an enhanced frontal negative response for concrete versus abstract word pairs, but only during RH presentation. Therefore, as a workaround of the lack of an abstract condition, the significant two-way interaction over the frontal ROI was broken down also along category (Bonferroni corrected α level = 0.0125). Responses evoked by literal expressions during LH versus RH presentation (Extended Data Fig. 2-2) were contrasted in the N700 time window, where Lucas et al. (2017) reported electrophysiological concreteness effects for novel expressions. Literals indeed evoked a greater negativity during RH than during LH presentation (β = –0.17, SE =* *0.06, *F*_(1,2913)_ = 8.63, *p *=* *0.003).

In order to address the potential explanation of the frontal effects based on the first constituents of the word pairs (as reported by Lucas et al., 2017), noun concreteness ratings were collected. Nouns were more concrete in literal than in metaphorical expressions (Table 1), yet a greater frontal negativity was evoked by metaphors relative to literals, therefore, an explanation of the frontal effects based on nouns can be excluded.

#### Metaphors split along median concreteness

To further investigate whether high-concreteness and low-concreteness metaphors are processed differently (Forgács et al., 2015), conditions were split along the median of concreteness (for examples, see Table 2). Both more abstract and more concrete metaphors were contrasted with high-concreteness literals, because these are the better exemplars of the concrete literal condition and thus could serve as a stricter baseline with respect to sensorimotor feature processing. Low-concreteness literals are merely lower on the concreteness scale and are neither truly abstract nor do they constitute a theoretically distinct category. Since the adjectives were not identical in these contrasts, their length and frequency were introduced to the models as a third element of a side × category interaction (they were included in the final models only if they significantly improved them). Adjective frequency information was retrieved from the Lexique database (New et al., 2004), and the logarithm of the subtitle-based word frequency was used, as it has a superior predictive power compared with other measures (Brysbaert et al., 2011). Since these latter two variables were of no theoretical interest and were included only to control for possible variance, their effect, as well as those of covariates not in interaction with category or side, are reported in detail only at http://osf.io/j45gt/.

**Table 2:** Examples of concrete and abstract, novel metaphorical and literal expressions

	Concrete		Abstract	
	tracteur bavard	talkative tractor	demande brisée	broken request
	narration mielleuse	honeyed narrative	affirmation biscornue	quirky statement
	agenda maigre	lean agenda	imprudence vide	empty recklessness
Metaphor	amende piquante	zesty fine	règlement tricoté	knitted settlement
	rumeur bouillante	boiling rumor	oubli dense	dense oblivion
	pensée enfumée	smoky thought	phrase graisseuse	fat sentence
	branche dansante	dancing branch	concept tuméfié	swollen concept
	figue croquante	crunchy fig	batelet usé	used dinghy
	chouquette délicieuse	delicious owl	soldat maigriot	skinny soldier
	mamie hurlante	screaming granny	catacombe fermée	closed catacomb
Literal	nectarine piquante	zesty nectarine	glaive décorée	decorated sword
	sucrerie pimentée	spicy candy	conserve empilée	stacked can
	marmelade coulante	flowing marmalade	artiste rassasiée	satiated artist
	lasagne bouillante	boiling lasagna	colloque vide	empty conference

Both conditions were split along their respective median concreteness values. Since less concrete literal expressions are not truly abstract and do not constitute a theoretically sound category, the best exemplars of the concrete literal condition, high concreteness literals served as baseline for comparison with both high-concreteness and low-concreteness (i.e., abstract) metaphors.

#### Low-concreteness (abstract) metaphors

First, low-concreteness metaphors were compared with high-concreteness literals in the 300- to 500-ms time window. In the final model [n400.ant.Ml.Lh.lmem = mixed(n400 ∼ category*side*bigram.frequency + category*side*concreteness + (1 + category|participant) + (1|item_nr), n400.ant.Ml.Lh.data)], there was a significant category × side × concreteness interaction (β = 0.29, SE =* *0.14, *F*_(1,2934)_ = 4.60, *p *=* *0.032). When it was broken down, there was no category main effect during LH presentation only when concreteness was not included, suggesting that it accounted for the effect. During RH presentation, a category × concreteness interaction was apparent (β = 0.61, SE =* *0.19, *F*_(1,243)_ = 9.87, *p *=* *0.002). When it was further broken down, concreteness affected ERP responses in opposite directions: high-concreteness literals followed a typical concreteness effect (β = –0.25, SE = 0.18), albeit non-significant (*p *=* *0.17), but low-concreteness metaphors showed a significant inverted effect: the less concrete (or more abstract) a metaphor was rated, the greater frontal negativity it evoked (β = 0.90, SE = 0.35, *F*_(1,90.7)_ = 6.49, *p *=* *0.013; Fig. 3).

Next, in the 700- to 1000-ms time window low-concreteness metaphors were compared with high-concreteness literals frontally. The final model [n700.ant.Ml.Lh.lmem = mixed(n700 ∼ category*side*concreteness + side*valence + (1 + category|participant) + (1 + side|item_nr), n700.ant.Ml.Lh.data)] showed a significant interaction between category × concreteness (β = 0.47, SE =* *0.15, *F*_(1,264)_ = 9.43, *p *=* *0.002). A category main effect and a category × side interaction was significant only when Concreteness was not included, which suggests that it accounted for the effect. When the former interaction was broken down by category, low-concreteness metaphors again showed an inverted concreteness effect (or abstractness effect; β = 0.84, SE =* *0.28, *F*_(1,94.6)_ = 9.02, *p *=* *0.003), while high-concreteness literals exhibited a non-significant (*p *=* *0.60) typical concreteness effect (β = –0.08, SE =* *0.15; Fig. 3).

#### High-concreteness metaphors

High-concreteness metaphors compared with high-concreteness literals in the 300- to 500-ms time window [n400.ant.Mh.Lh.lmem = mixed(n400 ∼ category*side*adjective.frequency + side*valence + (1|participant) + (1|item_nr), n400.ant.Mh.Lh.data)] revealed a main effect of category (β = –0.12, SE =* *0.05, *F*_(1,1672)_ = 5.06, *p *=* *0.025), and of side (β = 0.30, SE =* *0.14, *F*_(1,3026)_ = 4.49, *p *=* *0.034), and a tree-way interaction of category × side × adjective frequency interaction (β = 0.14, SE =* *0.06, *F*_(1,3029)_ = 5.29, *p *=* *0.022). When broken down along side, there was a significant difference between categories during LH presentation (β = –0.17, SE =* *0.07, *F*_(1,1159)_ = 5.72, *p *=* *0.017), but not during RH presentation (*p *=* *0.39).

Finally, high-concreteness metaphors were contrasted with high-concreteness literals in the 700- to 1000-ms time window. The final model [n700.ant.Mh.Lh.lmem = mixed(n700 ∼ category*side*adjective.frequency + side*valence + (1|participant) + (1|item_nr), n700.ant.Mh.Lh.data)] revealed a main effect of category (β = –0.13, SE =* *0.06, *F*_(1,3088)_ = 4.95, *p *=* *0.026), and a three-way interaction of category × side × adjective frequency (β = –0.13, SE =* *0.07, *F*_(1,3098)_ = 3.98, *p *=* *0.046). When broken down, there was no effect of condition during RH (*p *=* *0.83), only LH presentation, where metaphors evoked a greater negativity (β = –0.24, SE =* *0.08, *F*_(1,1512)_ = 8.93, *p *=* *0.003).

In sum, low-concreteness metaphors, relative to high-concreteness literals, evoked an abstractness effect during RH presentation in the 300- to 500-ms and bilaterally in the 700- to 1000-ms time windows: the less concrete (or more abstract) the metaphors were, the greater was the negativity they evoked. High-concreteness metaphors induced more negative responses frontally during LH presentation relative to high-concreteness literals in both time windows, just like in the main analyses.

## Discussion

The present study set out to explore neural responses to adjectives that refer to concrete, perceptual, physical experiences, when they serve as the figurative part of novel metaphorical expressions. The purpose of the experiment was two-fold: (1) to better understand the role of literal meaning and sensorimotor feature processing via the electrophysiological concreteness effect when concrete adjectives are meant in a metaphorical sense; and (2) to test whether the RH plays a unique role in figurative language processing.

Metaphorical expressions did not evoke a typical centroparietal N400 response relative to literal word pairs. Over the frontal ROI literal expressions elicited a electrophysiological concreteness effect: an enhanced negativity in the 700- to 1000-ms time window during RH relative to LH presentation. The least negativity was evoked by literals in the LH, where metaphors, compared to literals, evoked an enhanced frontal negativity in both the 300- to 500-ms and the 700- to 1000-ms time windows, a kind of effect that previously has not been reported with non-sentential stimuli. During RH presentation metaphors evoked the same level of negative amplitudes in the 300- to 500-ms time window as literals, however, these were the more negative the less concrete (or more abstract) the metaphors were, just the opposite of the typical concreteness effect. The frontal effect metaphors evoked in the 700- to 1000-ms time window was more negative bilaterally for increasingly more abstract metaphors. Neural responses to metaphors were not driven by figurativeness or imageability, but abstractness. RH presentation did not elicit a distinct response to metaphors, and the findings do not support an embodied account of semantic processing either, since sensorimotor feature processing, as reflected by a typical concreteness effects, was observed only for literal language.

The first important result is the lack of a typical centroparietal N400 effect for metaphors compared with literal expressions. Several studies reported an enhanced N400 effect when participants read novel metaphors, and most studies interpreted it as reflecting conceptual mappings and/or blends (Coulson and Van Petten, 2002, 2007; Arzouan et al., 2007; Lai et al., 2009; Goldstein et al., 2012; Rutter et al., 2012; Lai and Curran, 2013; Schneider et al., 2014; Tang et al., 2017; Rataj et al., 2018) or as the activation of literal senses (Pynte et al., 1996; Tartter et al., 2002; De Grauwe et al., 2010; Weiland et al., 2014). Such findings could have been artifacts of stimulus designs that did not take into consideration the novelty of metaphorical and literal control conditions. Metaphors might not elicit a typical N400 (Yang et al., 2013; Bardolph and Coulson, 2014) and might not be processed via blends, mappings, or literal senses.

The most remarkable outcome is a frontal response to metaphors both in the N400 and the N700 time windows during LH presentation. It conforms with a number of findings reporting LH effects for novel metaphors (Rapp et al., 2004, 2007; Diaz and Hogstrom, 2011; Forgács et al., 2012, 2014; Rutter et al., 2012). However, only Coulson and Van Petten (2007) reported specifically late left frontal negativities for metaphors, which they explained as a reduced frontal positivity because of low information selection demands. Instead of interpreting the effect as a reduced electrophysiological positivity, the frontal negativity for metaphors reported here could be an ERP response on its own right.

Its neural generators might be similar to or partially overlapping with those underlying left anterior negativities (LANs), which have been typically reported with sentential stimuli. It has been proposed that in the 300- to 450-ms time window LAN is sensitive to various morphosyntactic operations (Molinaro et al., 2015), for example, the violation of syntactic expectancy (Molinaro et al., 2011). A sustained LAN has been attributed to syntactic working memory load preceding syntactic integration (Fiebach et al., 2002), which can be evoked by function words (Neville et al., 1992; Kluender and Kutas, 1993) but also during thematic role assignment (King and Kutas, 1995). In their review, Federmeier and Laszlo (2009) concluded that a post-N400 frontal negativity could be related to meaning selection during ambiguity resolution. Novel metaphors plausibly require selecting an appropriate figurative sense of concrete adjectives, finding a link with the noun, and integrating the two into a unified representation. Developing the relational structure of the constituents and construing the properties of a combined representation could require syntactic-like operations and could easily pose working memory demands.

The frontal effects are clearly distinct from the electrophysiological concreteness effect. Lucas et al. (2017) found an enhanced frontal negativity in the N400 time window for abstract word pairs, which they interpreted as a positivity for concrete words pairs, driven by responses to first constituents. However, their results do not provide evidence for the additive nature of these effects, and an analysis of ERPs at the noun in the current study revealed no frontal concreteness effect, which could have explained later outcomes (Extended Data Figs. 2-2, 2-3). The lack of a difference between the metaphorical and literal conditions during RH presentation could imply an equal amount of sensorimotor feature processing. However, concreteness influenced amplitudes in the opposite direction for metaphors and literals. Literals evoked a typical concreteness effect, but metaphors evoked the greater frontal negativities the more abstract they were. This outcome replicates Forgács et al. (2015)’s result that abstract metaphors evoked a stronger N400 effect, not concrete ones. The ideas that novel metaphors rely on literal meanings (Bowdle and Gentner, 2005), that they are based on concrete source domains, involve mappings (Lakoff, 2014), or evoke sensorimotor processes (Gallese and Lakoff, 2005), beyond early automatic activations, which are not contested, did not receive empirical support. If metaphors do not transmit sensorimotor information via mappings to abstract target domains, and embodiment concerns only concrete literal language, its explanatory power for the conceptual system and cognition in general might be rendered rather limited.

Kousta et al. (2011) reported an abstractness effect in terms of reaction times, which challenged decades of concreteness effect research. The neural pattern of the electrophysiological abstractness effect contradicts Paivio (2007)’s dual coding theory, which suggests that all words activate a purely linguistic, amodal code in the LH, while concrete words activate an additional imagistic code in the RH. First, the concrete adjectives did not elicit a concreteness effect when used figuratively. Second, the abstractness effect was bilateral in the N700 time window, which questions the lateralized implementation of the two codes. Third, the fact that the RH produced a concreteness effect for literals in the N700 time window (replicating Huang et al., 2010; Lucas et al., 2017), while it contributed to an abstractness effect for metaphors in both time windows suggests that there is at least partial overlap between the neural generators of the verbal and imagistic codes, they are not lateralized in the two hemispheres. Further studies are necessary to specify the nature of the electrophysiological abstractness effect and its relation to sensorimotor feature processing.

One more notable finding of the present study is the left lateralization of the typical N400 both for novel metaphorical and literal stimuli (replicating Diaz and Hogstrom, 2011; Forgács et al., 2012, 2014; Davenport and Coulson, 2013). Both hemispheres are able to produce an N400 (Federmeier et al., 2005), which is indicative of a greater semantic memory retrieval effort (Kutas and Federmeier, 2011) or meaning activation (Molinaro et al., 2010). Prominent language lateralization models, such as the coarse semantic coding theory (Jung-Beeman, 2005) and the graded salience hypothesis (Giora, 2003), predict a greater RH activation during the comprehension of novel linguistic constructions. No such outcome was observed. Van Lancker Sidtis (2004)’s dual-process model can account for the current LH results: novel language, regardless of figurativeness, shall tax the LH because of effortful meaning construction. A curious finding is that emotional valence, which has been reported to evoke stronger responses when it is more negative (Altmann et al., 2012), did so in the current study when it was more positive, reflecting perhaps the joy of reading unfamiliar, creative expressions.

What processing steps could the electrophysiological response pattern indicate? The typical N400 response suggests a greater semantic activation (Molinaro et al., 2010; Kutas and Federmeier, 2011) in the LH for all novel word pairs. It is accompanied by an enhanced frontal N400 component in the LH for metaphors, which potentially reflects some sort of rule-based process (Molinaro et al., 2011, 2015), perhaps morphosyntactic/structural/conceptual combinatorics. This greater frontal negativity for metaphors is sustained during the 700- to 1000-ms time window, which could be related to (syntactic) working memory load (Fiebach et al., 2002), and/or ambiguity resolution and meaning selection (Federmeier and Laszlo, 2009). The frontal metaphor effects were sensitive to abstractness in the RH during the N400 time window and bilaterally during the N700 time window, which could be analogous to the conceptual manipulations proposed for the typical concreteness effect: the N400 for the activation (Gullick et al., 2013; Lucas et al., 2017) and the N700 for the conceptual integration (Barber et al., 2013), but in this case, of abstract properties. The suppression and enhancement of properties, which have been reported for metaphors (Gernsbacher et al., 2001) and for ambiguous words (Gergely and Pléh, 1994), could be reflected in these ERP responses. The adjective’s concrete, literal senses need to be suppressed and an appropriate, abstract, figurative sense needs to be activated, selected, and integrated into the representation of the expression.

The data are not inconsistent with Glucksberg (2003)’s categorization account, however, instead of describing the non-perceptual senses of metaphors in terms of superordinate categories and characterizing property selection along literal meaning (Glucksberg et al., 2001), abstractness might hold the key to figurative meaning. The abstract conceptual substitution model of metaphor comprehension (Forgács, 2014) proposes that the enhancement and suppression of conceptual properties (Gernsbacher et al., 2001) is carried out along the abstract-concrete dimension: not basic-level (or “literal”), but concrete properties are suppressed, and not superordinate (or “figurative”) but abstract properties are enhanced. From this narrower set of abstract senses, during the construction of metaphorical meaning, the contextually most relevant abstract property is substituted in the place of the figuratively meant word. The model does not require the construction of ad hoc categories (Glucksberg, 2003) or ad hoc concepts (Carston, 2010), not even metaphorical mappings (Lakoff and Johnson, 1980) or conceptual blends (Fauconnier and Turner, 1998), but suggests a unique form of ambiguity resolution for metaphors. Meaning filtering processes, similar to those employed for polysemous words (Murphy, 1997), could be used along abstractness, as a means of figurative “meaning making” (Bruner, 1990), a creative process of establishing, and not looking up, an interpretation. The theory moves beyond the literal-figurative distinction and can account for the reported abstractness effect as well: the more abstract the overall sense of the figurative expression is, the more effort could the search for the most appropriate abstract property require.

The present study reports of a frontal, negative going brain wave evoked by novel metaphors during LH presentation, which is sensitive to their abstractness, regardless of their meaningfulness, imageability, or figurativeness. The experiment did not find evidence for the RH theory of metaphor, for the involvement of the literal meaning of novel metaphors (Bowdle and Gentner, 2005), for the strong version of embodiment (Gallese and Lakoff, 2005; Lakoff, 2014), for some claims of Paivio (2007)’s dual coding theory, and for some prominent language lateralization models either (Giora, 2003; Jung-Beeman, 2005). Novel language appears to be processed by the LH and formulaic language by the RH (Van Lancker Sidtis, 2004), and metaphor appears to be no exception. Based on the data reported here, a novel picture of metaphor comprehension seems to emerge. The processing of figurative senses might not depend on concrete, sensorimotor features but on the semantic manipulation of abstract properties of words used metaphorically.

## Synthesis

Reviewing Editor: Satu Palva, University of Helsinki Decisions are customarily a result of the Reviewing Editor and the peer reviewers coming together and discussing their recommendations until a consensus is reached. When revisions are invited, a fact-based synthesis statement explaining their decision and outlining what is needed to prepare a revision will be listed below. The following reviewer(s) agreed to reveal their identity: Giosuè Baggio.

All concerns have been addressed
