# Supplementary material for: An Electrophysiological Abstractness Effect for Metaphorical Meaning Making
Source: eNeuro. 2020 Sep 10;7(5):ENEURO.0052-20.2020. doi: 10.1523/ENEURO.0052-20.2020 (PMC7559308; doi:10.1523/ENEURO.0052-20.2020)
Supplement: Extended Data Table 1-1 — Complete stimulus list. Every adjective was paired with two nouns to form both a metaphorical and a literal expression. Each participant saw either the literal or the figurative pairing for one particular adjective. Download Table 1-1, DOC file. [file enu-eN-NWR-0052-20-s08.doc]

**Table 1-1.** Complete stimulus list. Every adjective was paired with two nouns to form both a metaphorical and a literal expression. Each participant saw either the literal or the figurative pairing for one particular adjective.

| **Metaphor** | | **Literal** | |
| --- | --- | --- | --- |
| **Noun** | **Adjective** | **Noun** | **Adjective** |
| HARDIESSE | COULANTE | MARMELADE | COULANTE |
| CONTE | PÉRIMÉ | LITCHI | PÉRIMÉ |
| AUBAINE | CROQUANTE | FIGUE | CROQUANTE |
| SANTÉ | HURLANTE | MAMIE | HURLANTE |
| IRONIE | ODORANTE | BARQUE | ODORANTE |
| INDÉPENDANCE | DÉLICIEUSE | CHOUQUETTE | DÉLICIEUSE |
| EMPRUNT | IRRITANT | CACTUS | IRRITANT |
| DÉPENDANCE | SAVOUREUSE | MIRABELLE | SAVOUREUSE |
| SÉMINAIRE | JUTEUX | GRATIN | JUTEUX |
| DÉMARCHE | PIMENTÉE | SUCRERIE | PIMENTÉE |
| CÉLIBATAIRE | POIVRÉE | BISCOTTE | POIVRÉE |
| GÉNÉROSITÉ | FADE | GOYAVE | FADE |
| BONUS | PUANT | RADIS | PUANT |
| FILM | RASSIS | BONBON | RASSIS |
| INTUITION | MUSCLÉE | CHERCHEUSE | MUSCLÉE |
| ASTUCE | RÉCHAUFFÉE | LAITUE | RÉCHAUFFÉE |
| DÉCLARATION | CORPULENTE | SERVANTE | CORPULENTE |
| CONFRONTATION | OPAQUE | PAPYRUS | OPAQUE |
| MÉMORISATION | RIGIDE | MÉDAILLE | RIGIDE |
| GENTILLESSE | SUCCULENTE | GROSEILLE | SUCCULENTE |
| EXIGENCE | BOITEUSE | SAUTERELLE | BOITEUSE |
| PHRASE | GRAISSEUSE | HANCHE | GRAISSEUSE |
| ANALOGIE | IMMENSE | BICYCLETTE | IMMENSE |
| NOTATION | SVELTE | COUSINE | SVELTE |
| MYTHE | GLUANT | ÉTANG | GLUANT |
| IDÉE | OMBRAGÉE | BIBLIOTHÈQUE | OMBRAGÉE |
| ENSEIGNEMENT | ROUILLÉ | BAROMÈTRE | ROUILLÉ |
| DESTIN | CARBONISÉ | POIVRON | CARBONISÉ |
| BLAGUE | ÂCRE | GOUTTELETTE | ÂCRE |
| BRIMADE | POURRIE | BAUME | POURRIE |
| ALLÉGORIE | SALE | JAVELOT | SALE |
| FABULATION | GOÛTUE | ROQUETTE | GOÛTUE |
| CÉRÉMONIE | AMÈRE | PAPAYE | AMÈRE |
| INCOMPÉTENCE | SILENCIEUSE | ANTILOPE | SILENCIEUSE |
| CHANCE | AFFAMÉE | HIRONDELLE | AFFAMÉE |
| RÉFLEXION | SPACIEUSE | TRAPPE | SPACIEUSE |
| INTERACTION | RUINÉE | HORLOGE | RUINÉE |
| APOTHÉOSE | TENDRE | BLANQUETTE | TENDRE |
| SOCIÉTÉ | ÉBOURIFFÉE | FACTRICE | ÉBOURIFFÉE |
| DIPLOMATIE | LISSE | NAGEOIRE | LISSE |
| HUMEUR | CHAUFFÉE | VIENNOISERIE | CHAUFFÉE |
| FOI | CHATOYANTE | AMULETTE | CHATOYANTE |
| AFFAIRE | TREMBLANTE | CLARINETTISTE | TREMBLANTE |
| RÈGLEMENT | TRICOTÉ | OREILLER | TRICOTÉ |
| DIALECTIQUE | INERTE | FOURMI | INERTE |
| DYNAMISME | VOÛTÉ | KOALA | VOÛTÉ |
| RUMEUR | BOUILLANTE | LASAGNE | BOUILLANTE |
| JEUNESSE | GLISSANTE | BROUETTE | GLISSANTE |
| MENSONGE | TROUÉ | SUCRIER | TROUÉ |
| FRATERNITÉ | GROGNON | DENTISTE | GROGNON |
| ÉLOGE | VOMISSANT | OISEAU | VOMISSANT |
| ENGUEULADE | VIEILLIE | MARMITE | VIEILLIE |
| MINOIS | LAINEUX | CALEÇON | LAINEUX |
| PLAINTE | CABOSSÉE | LOUCHE | CABOSSÉE |
| CRITÈRE | BRUMEUX | CYCLONE | BRUMEUX |
| DÉTRESSE | SAIGNANTE | BRÛLURE | SAIGNANTE |
| ÉQUITÉ | DÉCHIRÉE | TULIPE | DÉCHIRÉE |
| REFLET | LIQUOREUX | YAOURT | LIQUOREUX |
| BOURSE | PLEURANTE | CHIENNE | PLEURANTE |
| FINALITÉ | ÉBLOUIE | PIANISTE | ÉBLOUIE |
| THÉORÈME | AGRÉABLE | NEUROLOGUE | AGRÉABLE |
| EXALTATION | FONDANTE | STALACTITE | FONDANTE |
| LIBERTÉ | SABRÉE | POIRE | SABRÉE |
| CHAPITRE | SUAVE | PAMPLEMOUSSE | SUAVE |
| ÉPISODE | RAYONNANT | LAMPADAIRE | RAYONNANT |
| ASSEMBLÉE | COLLANTE | PLUME | COLLANTE |
| MORALE | PERCÉE | BOTTINE | PERCÉE |
| COMPORTEMENT | PICOTANT | MELON | PICOTANT |
| REPRÉSENTATION | ENTASSÉE | CABINE | ENTASSÉE |
| SOUPÇON | GÂTÉ | POTIRON | GÂTÉ |
| MESSAGERIE | AGITÉE | LIMACE | AGITÉE |
| SOLITUDE | AFFINÉE | BRINDILLE | AFFINÉE |
| ÉNERGIE | MENOTTÉE | PATTE | MENOTTÉE |
| HISTOIRE | SCARIFIÉE | COMMERÇANTE | SCARIFIÉE |
| ADIEU | SCULPTÉ | TROMBONE | SCULPTÉ |
| PORTAIL | CHANTANT | PAPI | CHANTANT |
| CHAUSSETTE | ÉVISCÉRÉE | JUMENT | ÉVISCÉRÉE |
| COURAGE | RONFLANT | GORILLE | RONFLANT |
| TÂCHE | VALLONNÉE | PLAGE | VALLONNÉE |
| EMPATHIE | DORMANTE | COCCINELLE | DORMANTE |
| TEXTE | ASSÉCHÉ | PÂTURAGE | ASSÉCHÉ |
| PARCELLE | ÉTOUFFÉE | BREBIS | ÉTOUFFÉE |
| SONGE | FLUIDE | OMELETTE | FLUIDE |
| MONOLOGUE | DUVETEUX | FAUTEUIL | DUVETEUX |
| ENTENDEMENT | DYNAMIQUE | HÉRISSON | DYNAMIQUE |
| TRAFIC | ÉCARLATE | TRIEUR | ÉCARLATE |
| CHANT | BOSSELÉ | SOURCIL | BOSSELÉ |
| RELATION | ARRACHÉE | PLANCHETTE | ARRACHÉE |
| FOLIE | LIVIDE | NOURRICE | LIVIDE |
| CONSCIENCE | ONDULANTE | CRAVATE | ONDULANTE |
| PLAISANTERIE | RUGUEUSE | BABINE | RUGUEUSE |
| CAPRICE | DÉBORDANT | ABREUVOIR | DÉBORDANT |
| LOYAUTÉ | RADIEUSE | AMPOULE | RADIEUSE |
| INGÉRENCE | ÉLÉGANTE | THÉORICIENNE | ÉLÉGANTE |
| JUREMENT | SEC | BIGORNEAU | SEC |
| LIEN | FRIGORIFIÉ | ALIMENT | FRIGORIFIÉ |
| INTONATION | AMAIGRIE | BELETTE | AMAIGRIE |
| LEÇON | ÉTROITE | GOURDE | ÉTROITE |
| HARMONIE | ÉVANOUIE | GIRAFE | ÉVANOUIE |
| ÉLOCUTION | COLORÉE | MICHE | COLORÉE |
| PRINCIPE | FROISSÉ | CARTABLE | FROISSÉ |
| MÉCHANCETÉ | SOLIDE | TAPETTE | SOLIDE |
| ESPRIT | MAIGRIOT | SOLDAT | MAIGRIOT |
| SORTILÈGE | ROBUSTE | CICATRICE | ROBUSTE |
| ACCLAMATION | PARALYSÉE | CANTATRICE | PARALYSÉE |
| EXPLICATION | GRASSE | CHAUMIÈRE | GRASSE |
| INTELLIGENCE | PERFORÉE | MALLETTE | PERFORÉE |
| ROMANCE | PIPELETTE | MÉSANGE | PIPELETTE |
| RIVALITÉ | ASSOUPIE | PRÉSIDENTE | ASSOUPIE |
| PARFUM | DÉGARNI | ANANAS | DÉGARNI |
| GAÎTÉ | PÉTRIFIÉE | ACTRICE | PÉTRIFIÉE |
| APPÉTIT | ÉTIRÉ | MANCHE | ÉTIRÉ |
| LIGNAGE | EFFILOCHÉ | PAILLASSON | EFFILOCHÉ |
| STROPHE | ÉLIMÉE | PLINTHE | ÉLIMÉE |
| JOIE | DÉPLUMÉE | CANE | DÉPLUMÉE |
| SOUVENIR | BAVANT | JAGUAR | BAVANT |
| ATTITUDE | ÉTINCELANTE | TÉTINE | ÉTINCELANTE |
| RESPECTABILITÉ | SOUPLE | PINTADE | SOUPLE |
| ÉPITAPHE | ANIMÉ | RÉCEPTIONNISTE | ANIMÉ |
| ITINÉRAIRE | EMBUÉ | VASISTAS | EMBUÉ |
| ESCROQUERIE | ÉCLAIRÉE | MALLE | ÉCLAIRÉE |
| DÉFIANCE | FÉBRILE | LARVE | FÉBRILE |
| SEMAINE | MOUVANTE | GAZELLE | MOUVANTE |
| TRACTEUR | BAVARD | ÉPICIER | BAVARD |
| POÈME | LUXUEUX | CAILLOU | LUXUEUX |
| PLAIDOYER | USÉ | BATELET | USÉ |
| VIGILANCE | FERMÉE | CATACOMBE | FERMÉE |
| APOLOGIE | DÉCORÉE | GLAIVE | DÉCORÉE |
| BRUIT | CRASSEUX | FRUIT | CRASSEUX |
| CHANSON | DÉGRADÉE | AGRAFEUSE | DÉGRADÉE |
| QUESTION | TRESSÉE | HORTENSIA | TRESSÉE |
| MÉFIANCE | PUTRIDE | CASCADE | PUTRIDE |
| SCIENCE | BONDÉE | SOUPIÈRE | BONDÉE |
| CONCEPT | TUMÉFIÉ | MINISTRE | TUMÉFIÉ |
| OUBLI | DENSE | CAPUCHE | DENSE |
| PRÉCEPTE | ASSOURDI | CARNAVAL | ASSOURDI |
| EXAMEN | FLÉTRI | ARNICA | FLÉTRI |
| NOTE | BOSSUE | ÉPONGE | BOSSUE |
| IMPRUDENCE | VIDE | COLLOQUE | VIDE |
| ASSURANCE | FONDUE | GOUSSE | FONDUE |
| CÂLIN | TERNE | CHIOT | TERNE |
| CONNAISSANCE | GELÉE | MANIQUE | GELÉE |
| ARGUMENT | CONGELÉ | FEUTRE | CONGELÉ |
| AFFECTION | BROUILLÉE | COMPOTE | BROUILLÉE |
| INSTINCT | FULMINANT | CANON | FULMINANT |
| PARCOURS | HIRSUTE | ÉBOUEUR | HIRSUTE |
| FORÊT | CUITE | GRENADINE | CUITE |
| OPINION | INCLINÉE | TONG | INCLINÉE |
| VOLONTÉ | GRIMPEUSE | CREVETTE | GRIMPEUSE |
| STATUT | VAPOREUX | GÂTEAU | VAPOREUX |
| EXPOSÉ | MAGNÉTIQUE | CHAUSSON | MAGNÉTIQUE |
| CONFESSION | MOITE | COPIE | MOITE |
| BONHEUR | ASTHÉNIQUE | MAGASINIER | ASTHÉNIQUE |
| CONCOURS | FÉTIDE | PLANQUE | FÉTIDE |
| FUTUR | ÉPICÉ | KANGOUROU | ÉPICÉ |
| BONTÉ | GÉANTE | BLOUSE | GÉANTE |
| PROMESSE | GONFLÉE | RÉTINE | GONFLÉE |
| GOUVERNANT | TOXIQUE | MOJITO | TOXIQUE |
| ABSTINENCE | ÉNORME | ABRICOTIER | ÉNORME |
| AMENDE | PIQUANTE | NECTARINE | PIQUANTE |
| SINCÉRITÉ | CORROSIVE | ÉPICE | CORROSIVE |
| MENTALITÉ | ENDORMIE | DIRECTRICE | ENDORMIE |
| MALADRESSE | ÉCLATANTE | BALLE | ÉCLATANTE |
| HYPOTHÈSE | CASSABLE | BÛCHE | CASSABLE |
| AFFIRMATION | BISCORNUE | JACINTHE | BISCORNUE |
| CAMÉSCOPE | AVEUGLE | CALAMAR | AVEUGLE |
| RÉUSSITE | MUETTE | EMPLOYÉE | MUETTE |
| UNIVERSITÉ | FRAGMENTÉE | LUNETTE | FRAGMENTÉE |
| NARRATION | MIELLEUSE | GALETTE | MIELLEUSE |
| DÉCEPTION | GRIMPANTE | LIONNE | GRIMPANTE |
| IDÉOLOGIE | MÉLANGÉE | PÉPITE | MÉLANGÉE |
| MONARCHIE | SOUFFRANTE | AUTRUCHE | SOUFFRANTE |
| ONCLE | AIGRE | COCA | AIGRE |
| SALUTATION | DURE | CARAFE | DURE |
| ROUTINE | ÉPUISÉE | PHYSICIENNE | ÉPUISÉE |
| ÉTUDIANT | BUTINEUR | MOUSTIQUE | BUTINEUR |
| BRAVOURE | FRAGILE | GOURMETTE | FRAGILE |
| AMOURETTE | ERRANTE | BANQUIÈRE | ERRANTE |
| DISQUE | BÉGAYANT | VIEUX | BÉGAYANT |
| AGENDA | MAIGRE | BAMBIN | MAIGRE |
| DÉBAT | CLAQUANT | PORTIQUE | CLAQUANT |
| DEMANDE | BRISÉE | GRUE | BRISÉE |
| MÉDISANCE | FINE | CARPETTE | FINE |
| ESPOIR | TÉTANISANT | BONHOMME | TÉTANISANT |
| PENSÉE | ENFUMÉE | MOSQUÉE | ENFUMÉE |
| ÉPILOGUE | BRUYANT | CHIRURGIEN | BRUYANT |
| DOCTRINE | CORIACE | TANTINE | CORIACE |
| VÉRITÉ | EMPILÉE | CONSERVE | EMPILÉE |
| REMORD | LOURD | PANCRÉAS | LOURD |
| CONSOLATION | BALOURDE | MÉMÈRE | BALOURDE |
| VERSET | ENSOLEILLÉ | TIPI | ENSOLEILLÉ |
| COURTOISIE | GONFLANTE | PSYCHOLOGUE | GONFLANTE |
| FIDÉLITÉ | IGNOBLE | CAISSIÈRE | IGNOBLE |
| MAGAZINE | ÉLECTRISANT | CÂBLE | ÉLECTRISANT |
| CRAINTE | SUFFOCANTE | INHALATION | SUFFOCANTE |
| COURROUX | FIÉVREUX | CITOYEN | FIÉVREUX |
| PASSION | FRÉTILLANTE | CORNEILLE | FRÉTILLANTE |
| HAINE | RASSASIÉE | ARTISTE | RASSASIÉE |
| AVERTISSEMENT | TONIQUE | PARENT | TONIQUE |
| BRANCHE | DANSANTE | PERRUCHE | DANSANTE |
