# Supplementary figures and images for: An Electrophysiological Abstractness Effect for Metaphorical Meaning Making
Source: eNeuro. 2020 Sep 10;7(5):ENEURO.0052-20.2020. doi: 10.1523/ENEURO.0052-20.2020 (PMC7559308; doi:10.1523/ENEURO.0052-20.2020)

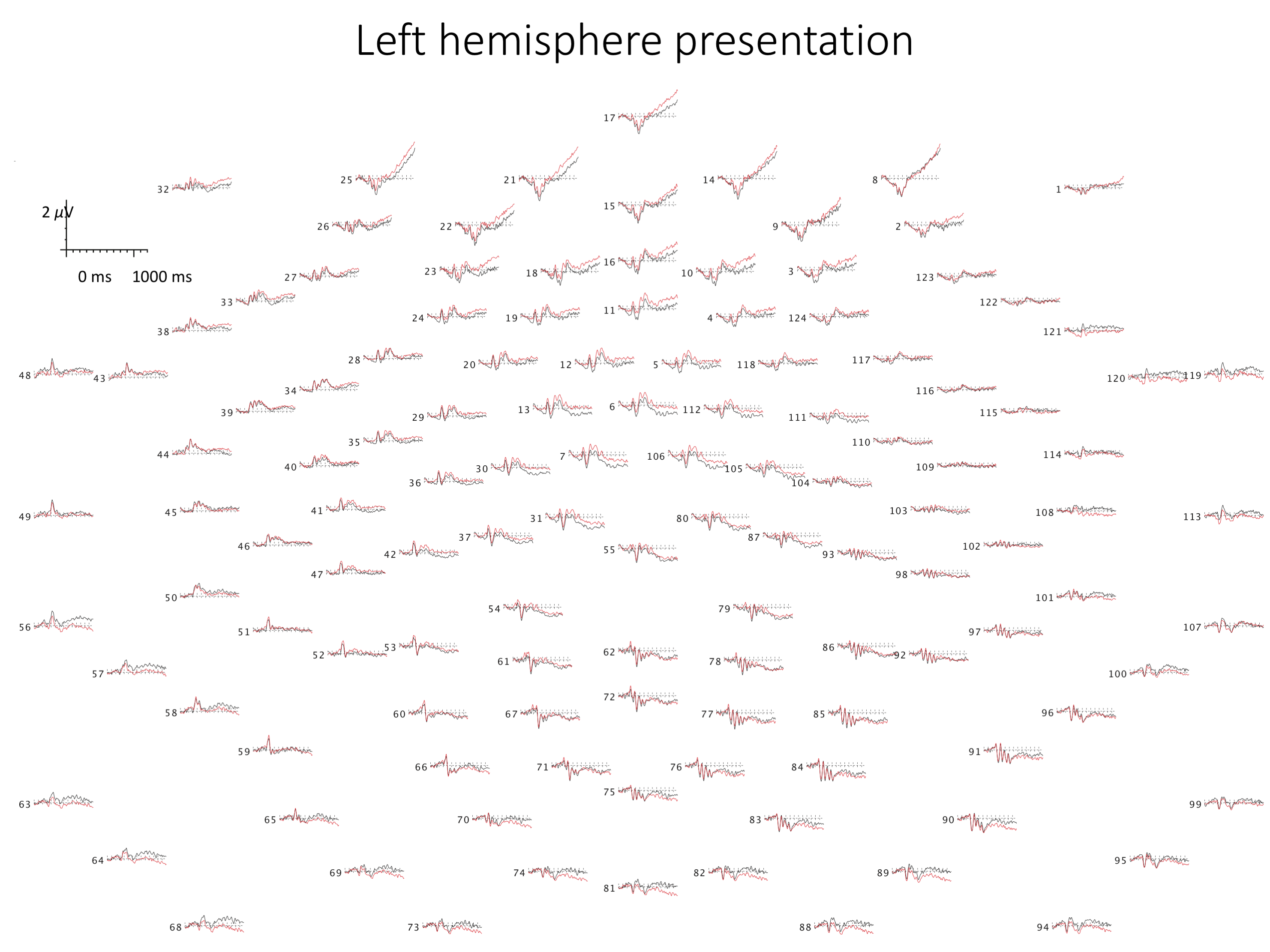

Supplement: Extended Data Figure 1-1 — Grand-average ERP plots for LH presentation over each electrode site. Negative is plotted upwards, frontal sites are above, parietal sites are below. Black line is the literal, red line is the metaphorical condition. A frontal negativity is apparent between 300–500 ms and 700–1000 ms as well. Download Figure 1-1, TIF file. [file enu-eN-NWR-0052-20-s02.tif]

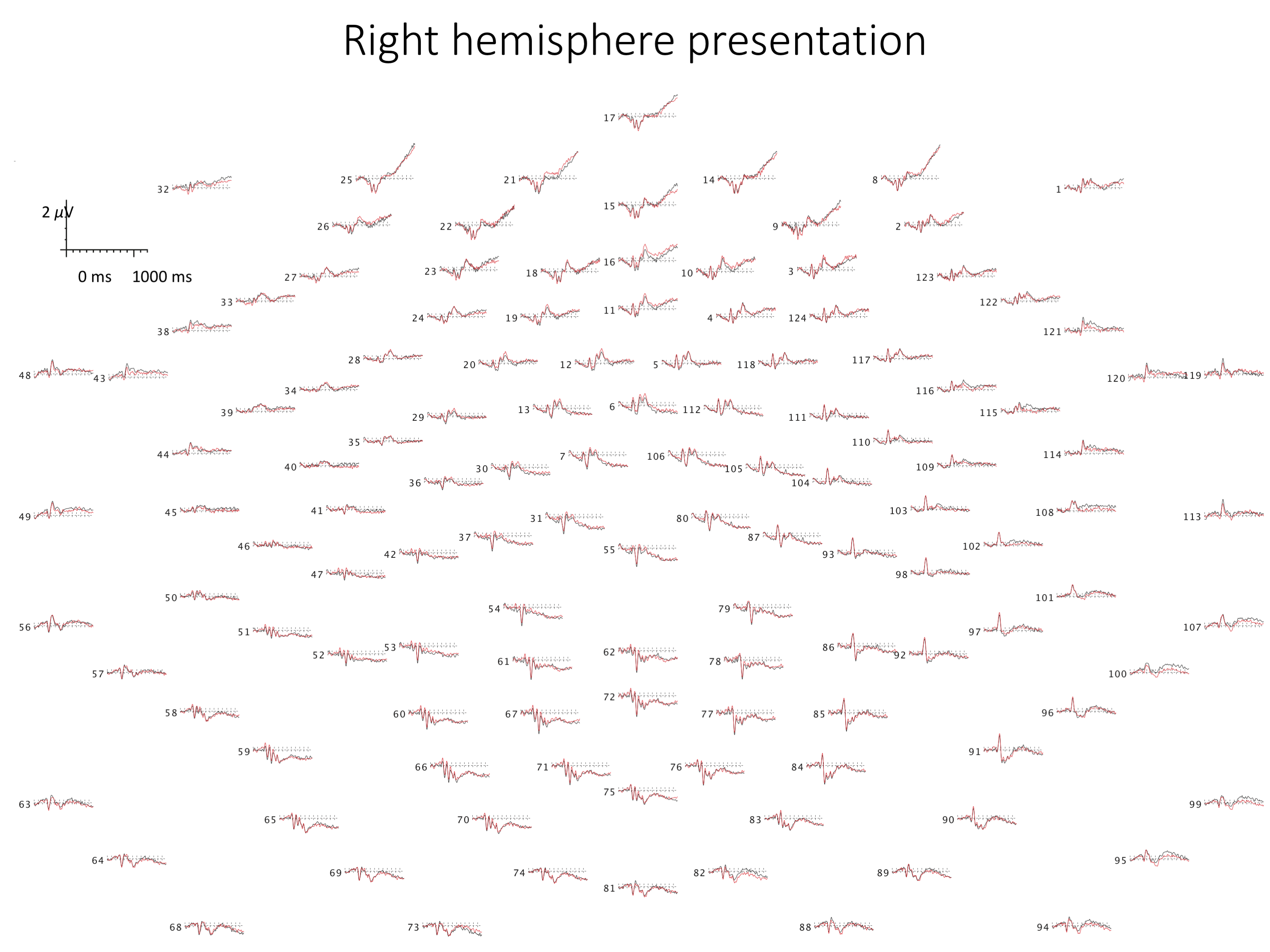

Supplement: Extended Data Figure 1-2 — Grand-average ERP plots for RH presentation over each electrode site. Negative is plotted upwards, frontal sites are above, parietal sites are below. Black line is the literal, red line is the metaphorical condition. No difference is apparent frontally between 300–500 or 700–1000 ms either. Download Figure 1-2, TIF file. [file enu-eN-NWR-0052-20-s03.tif]

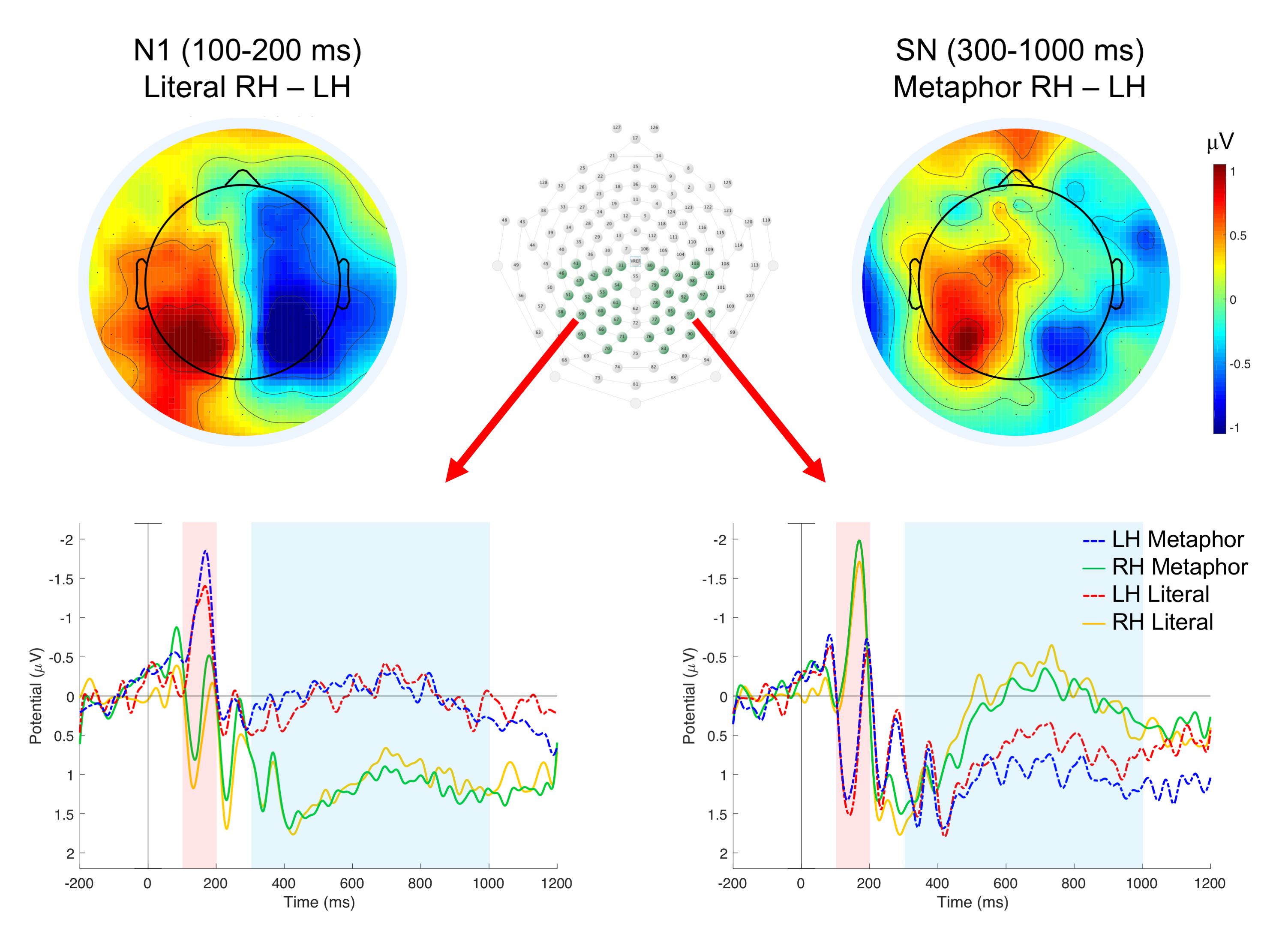

Supplement: Extended Data Figure 1-3 — Markers of lateralized processing. Topographical maps in the upper row show the difference of RH–LH presentation: cold colors indicate greater negativities for RH (left visual field), warm colors for LH (right visual field) presentation. Upper left represents the N1 for literal word pairs, and upper right the selection negativity (SN) for metaphors. Lower row ERP waveforms show responses on a left and a right exemplar electrode. Red shades indicate the 100- to 200 ms (N1) time window, blue shades the 300- to 1000-ms (SN) time window. Negative is plotted upwards. Download Figure 1-3, TIF file. [file enu-eN-NWR-0052-20-s04.tif]

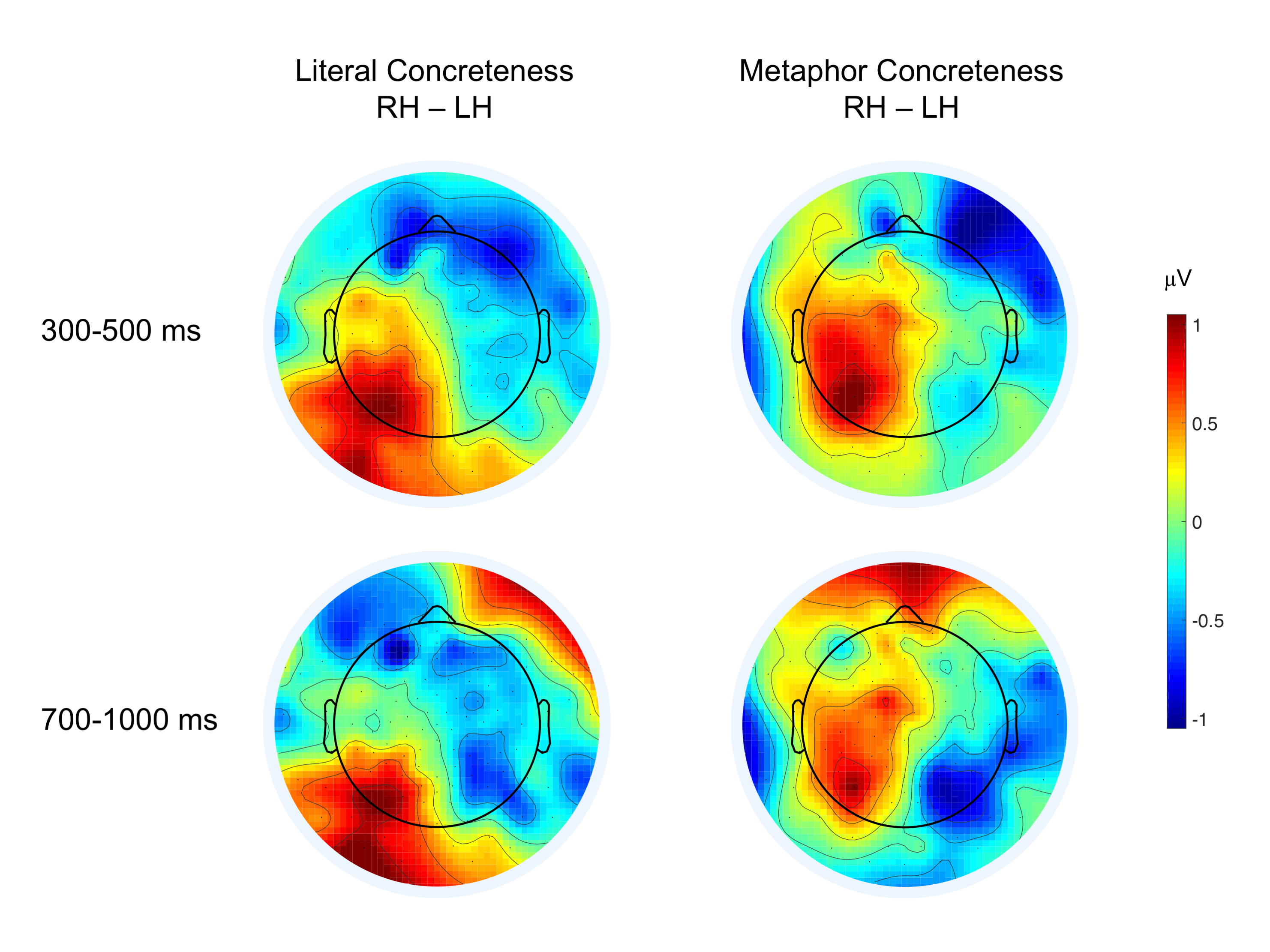

Supplement: Extended Data Figure 2-1 — Topographical maps of the frontal effects in the side contrast (RH–LH). Cold colors indicate greater negativities for RH (left visual field) presentation, warm colors for LH (right visual field) presentation. In the 300- to 500-ms time window neither metaphors (p = 0.11) nor literals (p = 0.13) were processed differently during lateralized presentation. In the N700 time window, literals evoked a greater negativity in the right than in the LH (β = –0.17, SE = 0.06, F (1,2913) = 8.63, p = 0.003), while metaphors evoked the same amount of brain electricity in both hemispheres (p = 0.059; Bonferroni corrected α-level = 0.0125). Download Figure 2-1, TIF file. [file enu-eN-NWR-0052-20-s05.tif]

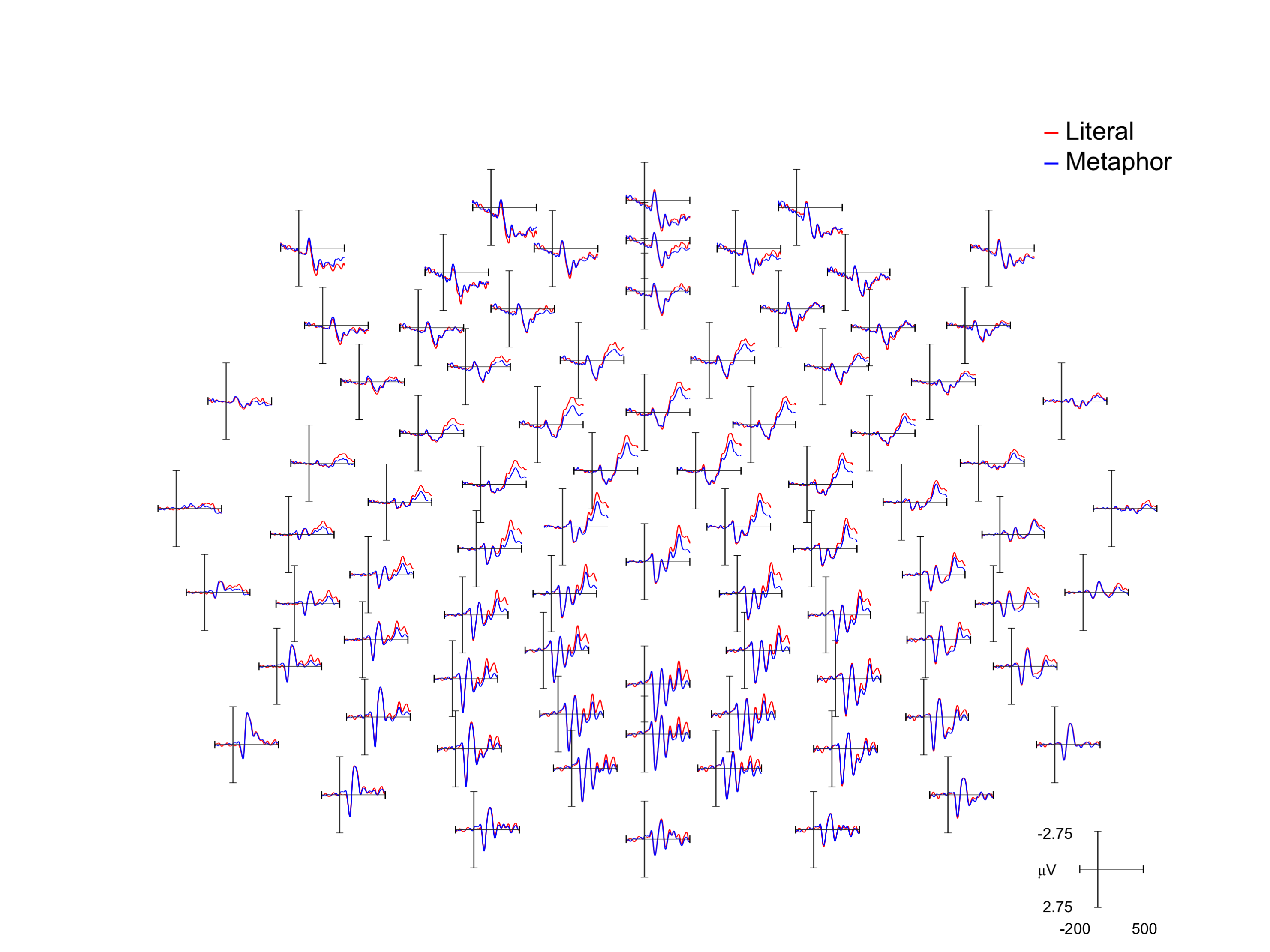

Supplement: Extended Data Figure 2-2 — Grand-average topo plots of ERPs at the prime word (noun) at each electrode site. Nouns were presented centrally for 200 ms followed by a blank screen jittered between 300 and 400 ms. Onset of visual word presentation is at 0, it is preceded by a 200-ms baseline and followed by 500-ms analysis window. Download Figure 2-2, TIF file. [file enu-eN-NWR-0052-20-s06.tif]

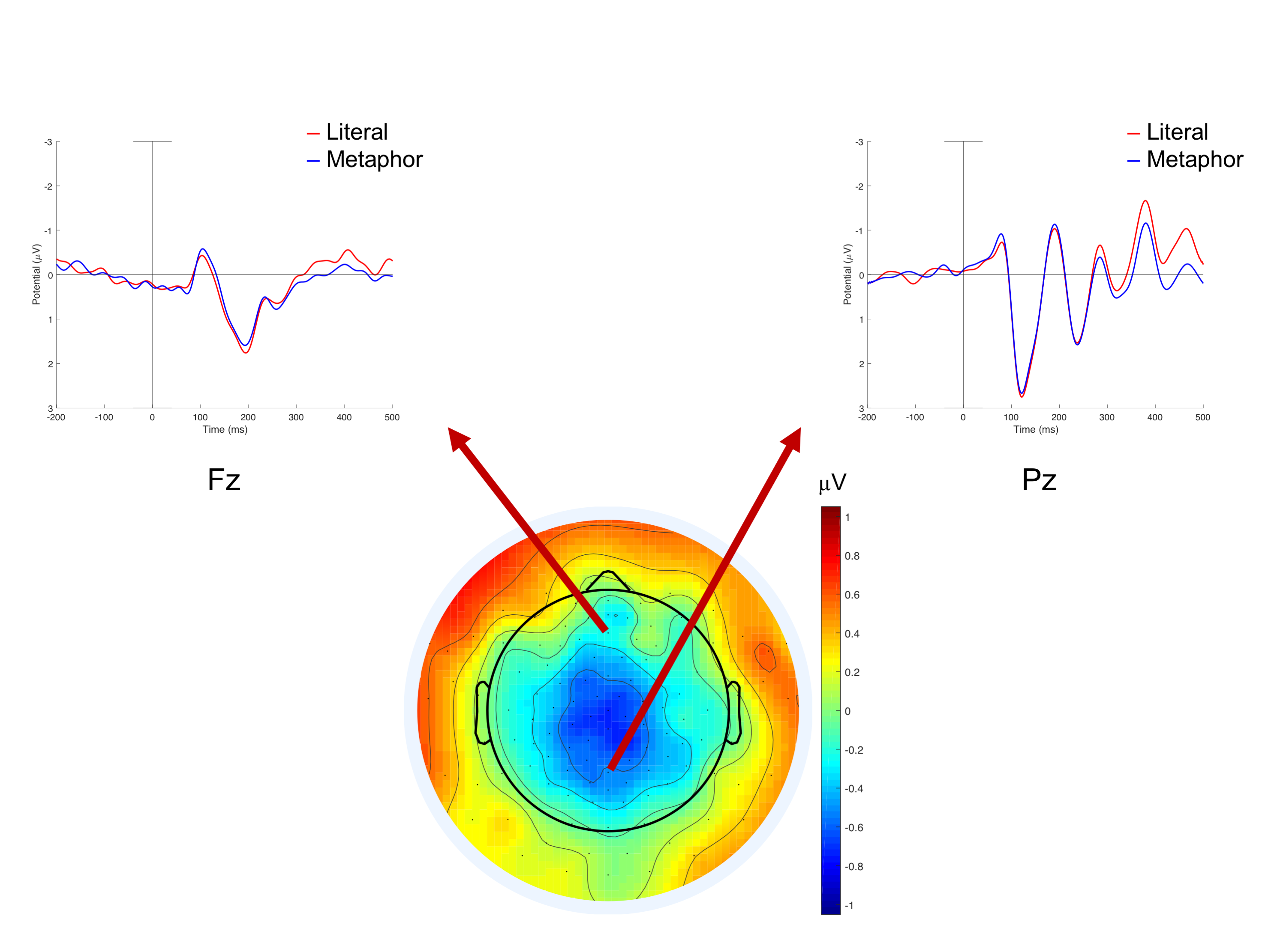

Supplement: Extended Data Figure 2-3 — Upper row shows ERP responses at two exemplar electrodes (Fz and Pz), lower row shows a topo map of ERPs in the N400 time window (300–500 ms) to the prime word (noun). Nouns evoked a typical N400 response, confirmed by a paired t test on participant averaged data over the parietal ROI (Mdiff = 0.39 μV, SDdiff = 0.41 μV), t (37) = 5.82, p < 0.001, 95% CI [0.25, 0.53 μV], Hedges’ g av = 0.43. Such an effect could be due to a variety of psycholinguistic variables (e.g., frequency, length, neighborhood size, etc.), since prime nouns were allowed to vary on properties, but target adjectives were fully controlled. Expressions in the literal and metaphorical conditions ended on the same adjectives, thus each participant saw the same set of target words. The response at the noun is not likely to have influenced outcomes at the adjective. If it influenced the baseline for adjectives, then responses to literals should have been artefactually reduced, which should have yielded an enhanced N400 response to metaphors (which was not the case). Download Figure 2-3, TIF file. [file enu-eN-NWR-0052-20-s07.tif]
